# 3,5-Dicaffeoylquinic acid protects H9C2 cells against oxidative stress-induced apoptosis via activation of the PI3K/Akt signaling pathway

**DOI:** 10.29219/fnr.v62.1423

**Published:** 2018-10-12

**Authors:** Yi-ming Bi, Yu-ting Wu, Ling Chen, Zhang-bin Tan, Hui-jie Fan, Ling-peng Xie, Wen-tong Zhang, Hong-mei Chen, Jun Li, Bin Liu, Ying-chun Zhou

**Affiliations:** 1 School of Traditional Chinese Medicine, Southern Medical University, Guangzhou, China; 2 Department of Traditional Chinese Medicine, Nanfang Hospital, Southern Medical University, Guangzhou, China; 3 Guangzhou Institute of Cardiovascular Disease, The Second Affiliated Hospital of Guangzhou Medical University, Guangzhou, China

**Keywords:** 3,5-dicaffeoylquinic acid, apoptosis, oxidative stress, PI3K/Akt pathway, cardiomyocyte

## Abstract

**Background:**

Oxidative stress-induced apoptosis plays an important role in the development of heart failure. 3,5-Dicaffeoylquinic acid (3,5-diCQA), a phenolic compound, has shown protective effects against oxidative stress in many diseases.

**Objective:**

The objective of this study was to investigate the anti-apoptosis potential of 3,5-diCQA in cardiomyocyte cells under oxidative stress and explore its underlying mechanisms.

**Design:**

A model of tert-butyl hydroperoxide (TBHP)-induced apoptosis in a cardiomyocyte cell line (H9C2) was established. Cell viabilities on cell lines were determined by 3-(4,5-dimethylthiazol-2-yl)-2,5-diphenyl tetrazolium (MTT) assay. The apoptosis was measured by hoechst33342 and propidium iodide (PI) fluorescent staining. PI (in red) stained the regions of cell apoptosis; Hoechet33342 (in blue) stained the nuclei. The Western blot was used to determine the expressions of related proteins such as p-PI3K: phosphorylated phosphatidylinositol-3-kinase (p-PI3K), phosphorylated Serine and Threonine kinase AKT (p-AKT), p-PTEN, Bcl-2, Bax, and caspase-3. Afterward, a PI3K inhibitor, LY294002, was applied to confirm the influence of the PI3K/Akt pathway on TBHP-treated cells of 3,5-diCQA. Then, H9C2 cells were pre-incubated with 3,5-diCQA alone to determine if the expression of activated PI3K/Akt signaling was mediated by 3,5-diCQA in H9C2 cells.

**Results:**

The results showed that TBHP resulted in an increase in cardiomyocyte apoptosis, whereas 3,5-diCQA treatment protected cells from TBHP-induced apoptosis in a dose-dependent manner. Moreover, 3,5-diCQA decreased expressions of Bax and caspase-3 but increased the phosphorylation levels of PI3K and Akt in TBHP-treated cells, which are the key molecules mediating cell survival, whereas phosphatase and tensin homologue deleted on chromosome 10 (PTEN) phosphorylation was unchanged. Importantly, pre-incubation with a PI3K inhibitor (LY294002) partly abolished the anti-apoptosis effects of 3,5-diCQA. Further, 3,5-diCQA enhanced the phosphorylation levels of PI3K and Akt in H9C2 cells directly, while LY294002 attenuated the effects of 3,5-diCQA on PI3K and Akt.

**Conclusion:**

This study suggested that 3,5-diCQA rescued myocardium from apoptosis by increasing the activation of the PI3K/Akt signaling pathway.

Heart failure (HF), the end-stage of various cardiovascular diseases, is a major cause of hospitalization and mortality worldwide, with an estimated death rate at first hospital admission of about 2–17% and more than 50% mortality within 5 years (1). One of the critical mechanisms of HF is oxidative stress-induced apoptosis, which occurs in myocardial infarction, atherosclerosis (AS) and ischemia or reperfusion. Therefore, inhibition of cardiomyocyte apoptosis is considered to be an effective strategy in the treatment of HF (2, 3).

Chlorogenic acids (CGAs), an important and biologically active dietary polyphenol found in fruit and plants such as coffee, cherries, and apples, show their potential anti-inflammatory and anti-oxidative effects in many diseases such as cardiovascular diseases and diabetes mellitus (4–6). 3,5-Dicaffeoylquinic acid (3,5-diCQA), also called *isochlorogenic acid A*, a major kind of CGA, is reported to be absorbed faster and metabolized more effectively than other CGAs (7). Previous studies have demonstrated that 3,5-diCQA exhibits protective effects against oxidative stress, inflammation, and gene mutations (8–10). Additionally, a number of studies indicate that 3,5-diCQA may have significant antihypertensive and anti-atherogenic effects in cardiovascular disease (11, 12). It has also been reported that 3,5-diCQA has protective effects on the heart (13). Moreover, treatment with 3,5-diCQA attenuated oxidative stress-induced apoptosis *in vivo* (14). Thus, 3,5-diCQA has become an attractive pharmacological treatment option for protecting cardiovascular cells from damage. Thus, in this study, we investigated the activity of 3,5-diCQA on cardiomyocyte apoptosis, which is one of the most important biological processes controlling HF, and explored the further mechanisms of 3,5-diCQA on regulating apoptosis in view of signal transduction.

In the cardiovascular system, the phosphatidylinositol 3-kinase (PI3K)/Akt pathway is closely related to regulation of cardiac development, angiogenesis, and apoptosis (15). Clinical studies found that a switch toward up-regulation of Akt is associated with the potential for failing myocardium in patients (16). It also reported that Akt activation in the left ventricle of AS patients undergoing coronary artery bypass grafting was beneficial in promoting both cardiomyocyte survival and its functional recovery (17). Experimental studies found that infarct size limitation and apoptosis inhibition were associated with phosphorylation of Akt, and these effects were blocked by the PI3K inhibitors LY294002 (18, 19). Moreover, activation of Akt reduced hydrogen peroxide-induced cell apoptosis in Ischemia/Reperfusion injury (20). In this study, an *in vitro* model of cardiomyocyte apoptosis was utilized to investigate whether the PI3K/Akt pathway was involved in the anti-apoptosis actions of 3,5-diCQA. The results of this study would shed more light on the mechanisms of 3,5-diCQA, which could potentially be used as a therapeutic agent for cardiovascular disease.

## Material and methods

### Reagents

3,5-DiCQA with over 98% purity was obtained from Chengdu Must Bio-technology Co. Ltd (Sichuan, China) and dissolved in dimethyl sulfoxide (DMSO) to make a stock solution. Tert-butyl hydroperoxide (TBHP) and Hoechst 33342/propidium iodide (PI) fluorescent staining kits were purchased from Sigma (St. Louis, MO, USA). Antibodies against glyceraldehyde-3-phosphate dehydrogenase, phospho-PI3K, phospho-PTEN, Akt, phospho-Akt, caspase-3, Bax, and Bcl-2 were procured from Cell Signaling Technology (Beverly, MA, USA). LY294002 was purchased from Haoyuan Chemexpress Co. Ltd (Shanghai, China).

### Cell culture and treatment

H9c2 cell line was purchased from the Cell Bank of Type Culture Collection of the Chinese Academy of Sciences (Lot No. GNR 5, Shanghai, China), and cells were cultured in Dulbecco's Modified Eagle Medium (DMEM) (Gibco, Grand Island, New York, USA) supplemented with 10% Fetal Bovine Serum (FBS) (Gibco) and 100 μg/mL penicillin and 100 μg/mL streptomycin (Gibco) at 37°C in a humidified atmosphere at 5% CO_2_ in air. Cells for the first five passages after cell thawing were utilized in the experiment. When cells were nearly 80–90% confluent, the medium was replaced with the DMEM supplemented with 2% FBS for another 12 h before experimental procedures. For experiments, cells were pre-incubated with different doses of 3,5-diCQA for 24 h and TBHP (75 μM) for another 4 h. For inhibitor experiments, cells were pre-incubated with PI3K inhibitor (LY294002, 25 μM) for 1 h as previously described (21) and then 3,5-diCQA and TBHP (75 μM). In our study, 3,5-diCQA should be firstly dissolved in DMSO and then the resulting solution in DMEM with the final concentration of DMSO less than 0.1%. As a toxic agent in our study, TBHP was diluted with DMEM as well.

### MTT assay

H9C2 cells were seeded in 96-well plates at a density of 1 × 10^4^ cells/well and incubated with test chemicals for the indicated time. After treatment, cell viability was assessed by MTT assay following the previous description (21).

### Hoechst33342/PI fluorescent staining

H9C2 cells were seeded in 24-well plates and pre-incubated with different stimulations. Following the treatment, cells were washed twice with cold Phosphate Buffer Saline (PBS) and stained with hoechst33342 for 20 min under dark conditions at 37°C and then with 1 μg/mL PI for another 5 min. The cell apoptosis index was quantified by averaging cell counts in three to four randomly selected fields per plate.

### Western blot assay

Expressions of proteins after different stimulations were detected by Western blot., Proteins were harvested according to the manufacturer’s instruction, and their contents were measured with a BCA protein assay kit (Beyotime, Shanghai, China). Then, proteins (30 μg) were separated by 10% Sodium Dodecyl Sulphate- PolyAcrylamide Gel Electrophoresis and transferred to a Polyvinylidene difluoride membrane (Millipore, Bedford, MA, USA). The membrane was blocked in 5% Bovine Serum Albumin for 2 h and then incubated with the following primary antibodies overnight at 4°C (p-PI3K 1:1,000, p-PTEN 1:1,000, Akt 1:2,000, p-Akt 1:1,000, caspase-3 1:2,000, Bax 1:1,000, Bcl-2 1:1,000, and GAPDH 1:3,000). Next, secondary antibodies conjugated to horseradish peroxidase (EARTH, San Francisco, CA, USA) were added to the membrane for 2 h and the results were quantified by Image J software (The National Institutes of Health and the Laboratory for Optical and Computational Instrumentation (LOCI, University of Wisconsin), Madison, USA).

### Statistical analysis

Data were presented as mean ± standard deviation. Statistical analysis was performed by one-way ANOVA followed by least significant difference test for comparisons between several groups. A value of *p* < 0.05 was considered statistically significant.

## Results

### Effects of 3,5-diCQA on TBHP-induced injury in H9C2 cells

**Fig. 1:**
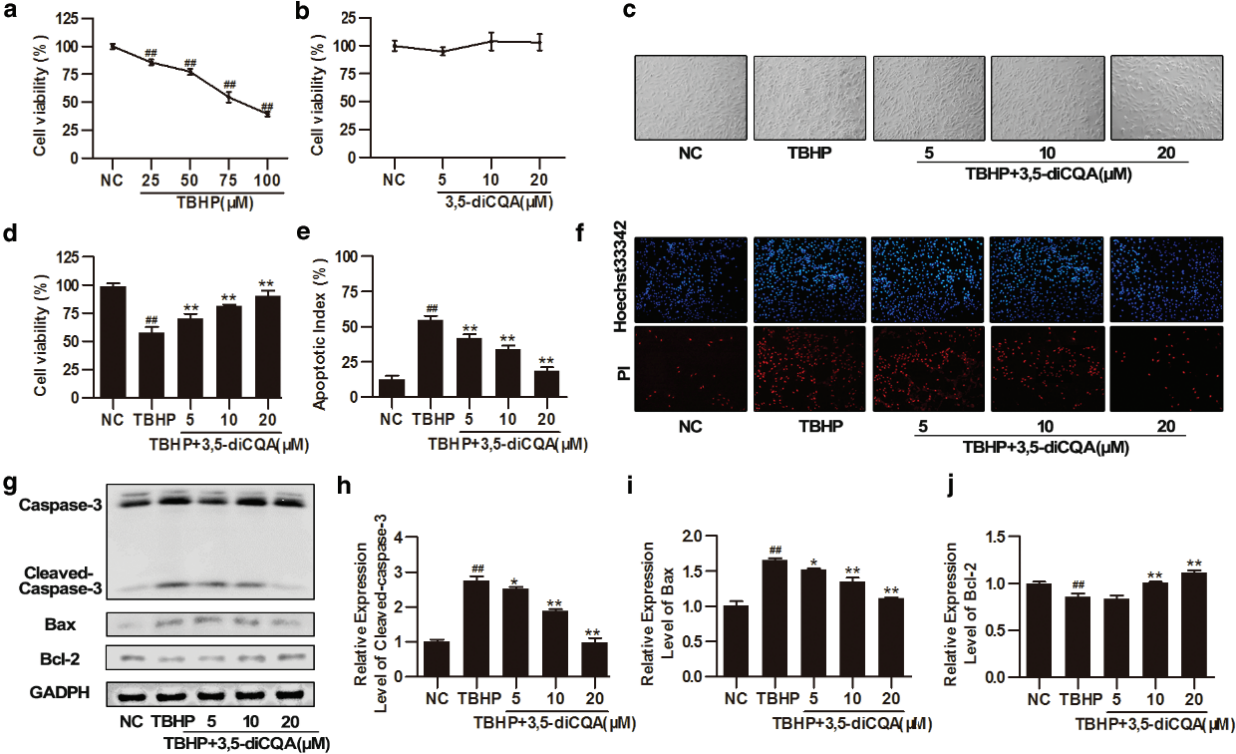
Effects of 3,5-dicaffeoylquinic acid (3,5-diCQA) on tert-butyl hydroperoxide (TBHP)-induced injury in H9C2 cells. H9C2 cells were pre-incubated with the indicated doses of 3,5-diCQA (5, 10, and 20 μM) for 24 h and then stimulated with TBHP (75 μM) for 4 h. Cell viability of H9C2 cells was measured by MTT assay. (a) Cytotoxicity of TBHP (*n* = 6); (b) cytotoxicity of 3,5-diCQA (*n* = 6); (c) representative bright field images of H9C2 cells; (d) cell viability of 3,5-diCQA on TBHP-induced apoptosis (*n* = 6); (e and f) percentage of apoptotic cells of H9C2 determined by Hoechst 33342/ PI staining (*n* = 3); (g) Western blotting of apoptosis-related proteins including cleaved caspase-3, Bax, and Bcl-2 and their quantification (h through j) (*n* = 3). Results were expressed as means ± SD. #*p* < 0.05, ##*p* < 0.01 vs. normal control (NC); *p < 0.05, **p < 0.01 vs. TBHP group.

As a cytotoxic agent, TBHP reduced cell vitality in a dose-dependent manner within the range of 25–100 μM; 75 μM TBHP decreased H9C2 viability to 54.61 ± 4.78% (*p* < 0.05), which was used to establish an apoptotic model in subsequent experiments (Fig. 1a). Additionally, to evaluate the cytotoxicity of 3,5-diCQA, H9C2 cells were pre-incubated with 3,5-diCQA (5, 10, and 20 μM) for 24 h. The results showed that cell survival in these groups was similar to that in the control group (*p* > 0.05), indicating that 3,5-diCQA has no toxicity toward H9C2 cells (Fig. 1b). To examine the protective effect of 3,5-diCQA against TBHP-induced injury, H9C2 cells were pre-incubated with indicated doses of 3,5-diCQA (5, 10, and 20 μM) for 24 h and then cells were stimulated with 75 μM TBHP for another 4 h. Cell viability was detected using the MTT assay, cell apoptotic index was measured by Hoechst 33342/PI fluorescent staining, and the expression of apoptosis-related proteins was detected by Western blotting. MTT assay showed that exposure to TBHP reduced cell viability to 57.61% and resulted in cell shrinkage and alterations of cell shape, whereas pre-incubation with different concentrations of 3,5-diCQA (5, 10, and 20 μM) not only restored cell morphologies but also improved cell survival by 12.48%, 23.74% and 32.92%, respectively (*p* < 0.05; Fig. 1c and d). Hoechst 33342/PI fluorescent staining was also performed to detect the number of apoptotic cells. The red regions (PI) represent apoptosis and the blue regions (Hoechst 33324) represent the nuclei. Consistent with the MTT assay results, most cells were stained red and showed features characteristic of apoptosis after TBHP incubation. In contrast, 3,5-diCQA (5, 10, and 20 μM) attenuated these changes in H9C2 cells in a dose-dependent manner (*p* < 0.05; Fig. 1e and f). To determine if the levels of apoptosis-related proteins such as caspase-3, Bax, and Bcl-2 changed, we performed Western blotting. Cleaved caspase-3, as the final effector of apoptosis, was up-regulated after TBHP incubation, while 3,5-diCQA resulted in a dose-dependent reduction of cleaved caspase-3 expression (*p* < 0.05; Fig. 1g and h). Meanwhile, 3,5-diCQA reduced the expression level of the pro-apoptotic protein Bax but increased the expression level of anti-apoptotic protein Bcl-2, indicating that the ratio of Bcl-2 to Bax was enhanced by 3,5-diCQA compared to TBHP (*p* < 0.05; Fig. 1g through j). All these results indicated that TBHP-induced apoptosis in H9C2 cells and 3,5-diCQA could inhibit such apoptotic activity of TBHP.

### Effects of 3,5-diCQA on PI3K/Akt signaling pathway in H9C2 cells exposed to TBHP

**Fig. 2:**
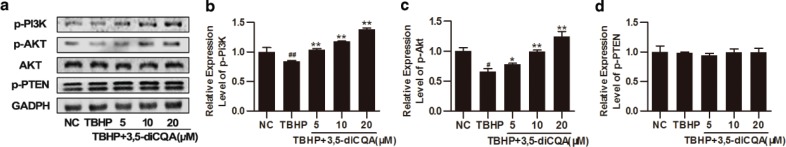
Effects of 3,5-diCQA on phosphatidylinositol 3-kinase (PI3K)**/**Akt signaling pathway in H9C2 cells exposed to TBHP. H9C2 cells were pre-incubated with the indicated dose of 3,5-diCQA (5, 10, and 20 μM) for 24 h and then stimulated with TBHP (75 μM) for 4 h. (a) Western blot was performed to demonstrate the expression of p-PI3K, p-Akt, and p-PTEN, and densities of the bands were quantified by densitometry analysis (b through d) (*n* = 3). Data were shown as mean ± SD. #*p* < 0.05, ##*p* < 0.01 vs. normal control (NC); *p < 0.05, **p < 0.01 vs. TBHP group.

The PI3K/Akt signal pathway plays an important role in cardioprotection and survival (15). In order to explore the mechanisms of 3,5-diCQA in protecting H9C2 from TBHP-induced injury, expressions of key molecules in the PI3K/Akt signaling pathway were measured by Western blot. Akt is activated by PI3K in a phosphorylate-dependent manner and termination of PI3K signaling is primarily achieved by the phosphatase PTEN. As Fig. 2 shows, compared with the control groups, the reductions of p-PI3K and p-AKT by TBHP was remarkable (*p* < 0.05). However, the results showed increasing expressions of p-PI3K and p-AKT by 3,5-diCQA pre-incubation when compared with TBHP (*p* < 0.05), while 3,5-diCQA had no significant effect on the expression of p-PTEN (*p* > 0.05). These results suggest that 3,5-diCQA promotes the activation of PI3K/Akt signaling in cells exposed to TBHP.

### Effects of 3,5-diCQA in TBHP-induced injury of H9C2 cells under inhibition of PI3K/Akt signaling pathway

**Fig. 3:**
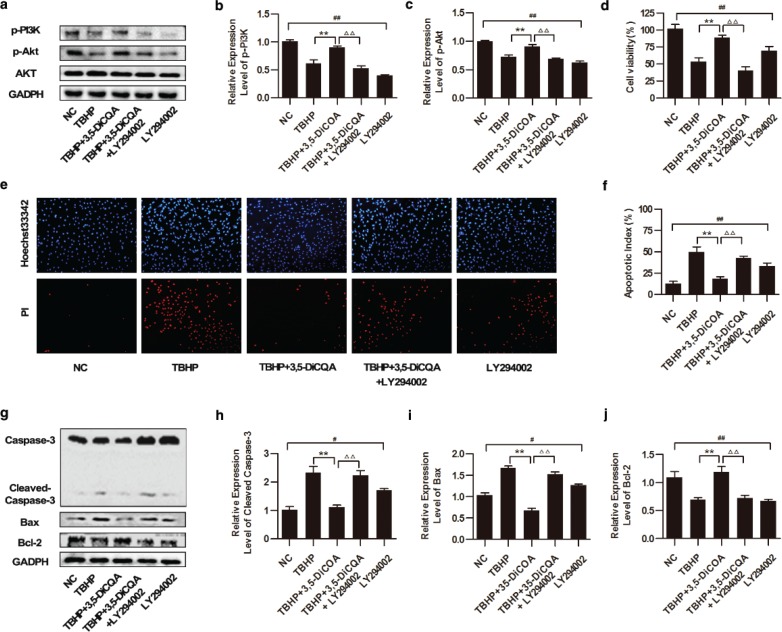
Effects of 3,5-diCQA on TBHP-induced injury of H9C2 cells under inhibition of the PI3K**/**Akt signaling pathway. LY294002 was applied to inhibit the activation of the PI3K/Akt signaling pathway in this section. H9C2 cells were pre-incubated with 25 μM LY294002 for 1 h, and 20 μM 3,5-diCQA for another 24 h and then 75 μM TBHP. (a) Western blotting of the PI3K/Akt signaling and the fold activation data analysis (b and c) (*n* = 3); (d) cell viability of H9C2 determined by MTT assay (*n* = 6); (e and f) Percentage of apoptotic cells of H9C2 determined by Hoechst 33342/PI staining (*n* = 3); (g) Western blotting of apoptosis-related proteins including cleaved caspase-3, Bax and Bcl-2 and the fold activation data analysis (h through j) (*n* = 3). Data were shown as mean ± SD. #*p* < 0.05, ##*p* < 0.01 vs. normal control (NC); *p < 0.05, **p < 0.01 vs. TBHP group; ^Δ^*p* < 0.05, ^ΔΔ^*p* < 0.01 vs. 3,5-DiCQA + TBHP group.

To confirm the influence of the PI3K/Akt pathway on the cytoprotection of 3,5-diCQA, the effects of a PI3K-inhibitor, LY294002, were next examined. Cells were pre-incubated with 25 μM LY294002 for 1 h, co-incubated with 20 μM 3,5-diCQA for another 24 h, and then finally incubated with 75 μM TBHP. The levels of p-PI3K and p-AKT were measured by Western blotting. It was found that these proteins were induced by 3,5-diCQA supplementation in cells exposed to TBHP (*p* < 0.05), while LY294002 addition significantly suppressed the expressions of p-PI3K and p-AKT, resulting in 37.29% and 21.64% fold protein reduction, respectively. In addition, LY294002 alone suppressed the phosphorylations of both PI3K and AKT significantly compared with the normal control (NC) group (*p* < 0.05; Fig. 3a through c). Next, to further verify whether the anti-apoptosis effect of 3,5-diCQA was blocked by LY294002 addition, cell viability, apoptotic index and the expressions of apoptosis-related proteins were detected. MTT results showed that the increased cell viability of 3,5-diCQA was impeded by LY294002 from 89.11±3.25% to 40.52 ± 5.71% in TBHP-treated cells (*p* < 0.05; Fig. 3d). Meanwhile, Hoechst 33342/PI fluorescent staining demonstrated that the addition of LY294002 increased the cell apoptosis index by 24.43% as compared to that with the 3,5-diCQA treatment (*p* < 0.05; Fig. 3e and f). Consistently, addition of LY294002 exerted a similar effect on increasing both caspase-3 cleavage and Bax expressions, resulting in 111.9% and 85.21% fold protein increment, respectively, whereas it decreased Bcl-2 expression by 46.49% compared to 3,5-diCQA treatment (*p* < 0.05, Fig. 3g through j). Additionally, LY294002 alone also induced apoptosis of H9C2 cells concomitant with the increase of both the Bax/Bcl-2 ratio and caspase-3 cleavage compared with the NC group (*p* < 0.05). All the results suggested that inhibition of PI3K/Akt signaling pathway partly blocked the anti-apoptosis effect of 3,5-diCQA.

### Effects of 3,5-diCQA on the expression of activated PI3K/Akt signaling mediators in H9C2 cells

**Fig. 4:**
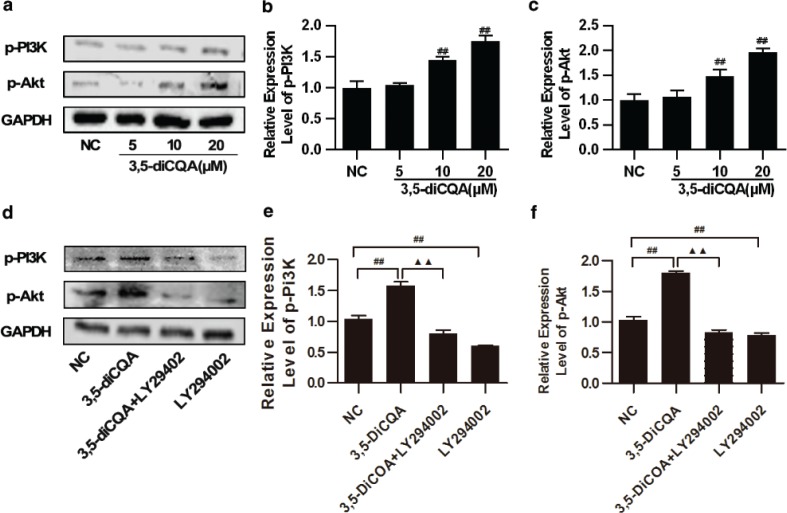
Effects of 3,5-diCQA on the expression of activated PI3K**/**Akt signaling mediators in H9C2 cells. H9C2 cells were incubated with 3,5-diCQA (5, 10, and 20 μM) for 24 h. LY294002 was used in inhibitory experiments and H9C2 cells were pre-incubated with 25 μM LY294002 for 1 h, and 20 μM 3,5-diCQA for another 24 h. The indicated proteins were detected by Western blot. (a) Representative images of p-PI3K and p-Akt expression and their quantification (b and c); (d) expression of p-PI3K and p-Akt in presence of LY294002 and their quantification (e and f). The results were expressed as means ± SD (*n* = 3). #*p* < 0.05, ##*p* < 0.01 vs. normal control (NC); ^▲^*p* < 0.05, ^▲▲^*p* < 0.01 vs. 3,5-DiCQA group.

Next, to further study the effects of 3,5-diCQA on the expression of activated PI3K/Akt signaling, H9C2 cells were pre-incubated with 3,5-diCQA (5, 10, 20 μM) for 24h and p-PI3K and p-Akt were detected. The results of the Western blot showed that 3,5-diCQA promoted phosphorylations of PI3K and Akt dose-dependently (*p* < 0.05, Fig. 4a through c). LY294002 alone was also applied here, which significantly reduced the phosphorylation levels of PI3K and Akt, compared with the NC group (*p* < 0.05). LY294002 also significantly reduced the increased phosphorylation levels of PI3K and Akt of 3,5-diCQA by 77.21% and 96.69%, respectively (*p* < 0.05; Fig. 4d through f). These results demonstrated that activation of PI3K/Akt was involved in the anti-apoptosis action of 3,5-diCQA.

## Discussion

In this study, we investigated the protective effects and mechanisms of 3,5-diCQA against oxidative stress-induced H9C2 apoptosis. Our study showed that the anti-apoptotic effects of 3,5-diCQA are associated with the activation of the PI3K/Akt pathway.

Cardiomyocyte apoptosis plays a critical role in the aetiology and pathogenesis of HF and oxidative stress is one of the major factors that induce cardiomyocyte apoptosis (1, 22). Inhibition of cardiomyocyte apoptosis contributes to ameliorating the failing myocardium and is the basis for therapy for HF (23, 24). 3,5-diCQA, a common derivative of coffee, was found to protect neuronal cells in neurodegenerative diseases implicated with oxidative stress (25,26). A previous study also found that 3,5-diCQA possesses a cytoprotective effect against H_2_O_2_-induced oxidative stress on SH-SY5Y cells (27). However, the effects of 3,5-diCQA on oxidative stress-induced apoptosis of H9C2 cells are not clear. Therefore, in this study, we induced the oxidative stress injury in H9C2 by using TBHP, which is one of the most common oxidizing agents in experiments (28). In this study, we found that 3,5-diCQA showed a protective effect on H9C2 cells with an improvement of cell viability in a dose-dependent manner, which is in agreement with previous researches (26, 27). These results demonstrated that 3,5-diCQA inhibits oxidative stress-induced apoptosis in H9C2. The H9C2 cell line of embryonic rat cardiomyocytes is derived from embryonic BD1X rat heart tissue, and HL-1 cells are a popular model for use as a cardiomyocyte cell line (29, 30). At the beginning of this study, we compared the backgrounds of HL-1 and H9C2. We found both of these cell lines are immortalized cells with a cardiac phenotype, and both are widely used for the analysis of cardiac oxidative stress injury. However, it has been reported that H9C2 cells are more similar to primary cardiomyocytes than HL-1 cells with regard to energy metabolism patterns, such as cellular ATP levels, bioenergetics, metabolism, function, and morphology of mitochondria, and were significantly more sensitive to oxidative stress than HL-1 cells (31). Thus, we chose the H9C2 cell line to establish a cell model. In addition, performing the experiment on only one cell line is a limitation of this study.

When the myocardium is subjected to oxidative stress, the metabolic and functional characteristics of the mitochondria change and the mitochondria-mediated intrinsic apoptosis pathway is initiated. The by-products of oxidative stress such as reactive oxygen species react directly with membrane lipids and proteins, causing mitochondrial dysfunction and changes of apoptotic proteins, including the Bcl-2 homology domain 3-domain interaction between Bax and Bcl-2, release of cytochrome c and final activation of caspases, notably caspase-3, all of which induce apoptosis in cells. Furthermore, a lower ratio of Bcl-2 to Bax is associated with higher apoptosis in cells (22, 32). It has been reported that 3,5-diCQA attenuated caspase-3 activation induced by H_2_O_2_, causing an increase in survival of SH-SY5Y cells *in vitro* (14). Consistently, 3,5-diCQA may prevent neuronal apoptosis through the repression of apoptotic signaling molecules such as Bax *in vivo* (26). In this study, we found that 3,5-diCQA was indeed able to reduce apoptosis induced by TBHP in H9C2 cells by improving the ratio of Bcl-2 to Bax and descending cleaved caspase-3, indicating that 3,5-diCQA inhibits apoptosis by suppression of the mitochondria-mediated intrinsic apoptosis pathway.

The PI3K/Akt pathway involves the process of growth and survival in cells. It has been established that activation of the PI3K/Akt pathway by human growth factor (HGF) appears to be necessary for the anti-apoptotic effects of HGF in cardiomyocytes (32). In this study, we determined whether 3,5-diCQA performed the anti-apoptotic actions through activation of PI3K/Akt pathway. We found that the expressions of PI3K and Akt were significantly decreased by TBHP, whereas 3,5-diCQA dose-dependently activated the expressions of PI3K and Akt. Thus we hypothesized that the anti-apoptotic action is related to the activation of the PI3K/Akt pathway. To examine this hypothesis, a specific PI3K-inhibitor, LY294002 was applied for the next experiments. We found that LY294002 abolished the anti-apoptotic actions of 3,5-diCQA, causing low cell viability and high apoptosis, which was similar to those in TBHP. Notably, treatment cells with 3,5-diCQA alone activated PI3K and Akt directly, which were inhibited by LY294002 as well. Our results were in agreement with a previous study that 3,5-diCQA exhibits a neuroprotective effect through protection of mitochondrial activities and activation of Akt (26).

## Conclusion

In summary, we found that 3,5-diCQA could inhibit apoptosis by increasing the activation of the PI3K/Akt signaling pathway in TBHP-treated H9C2 cells. Our data provides new insights into the mechanisms of 3,5-diCQA in anti-apoptotic action under oxidative stress *in vitro*.
